# The PGRMC1 Antagonist AG-205 Inhibits Synthesis of Galactosylceramide and Sulfatide

**DOI:** 10.3390/cells10123520

**Published:** 2021-12-13

**Authors:** Lihua Wang-Eckhardt, Ivonne Becker, Matthias Eckhardt

**Affiliations:** Institute of Biochemistry and Molecular Biology, Medical Faculty, University of Bonn, 53115 Bonn, Germany; eckhardt@institut.physiochem.uni-bonn.de (L.W.-E.); ivonne22@uni-bonn.de (I.B.)

**Keywords:** AG-205, galactosylceramide, UDP-galactose: ceramide galactosyltransferase, PGRMC1, PGRMC2, sphingolipids, sulfatide

## Abstract

Sulfatide synthesis in the human renal cancer cell line SMKT-R3 was strongly inhibited in the presence of low µM concentrations of AG-205, a progesterone receptor membrane component 1 (PGRMC1) antagonist. This was also the case in Chinese hamster ovary (CHO) cells stably transfected with UDP-galactose: ceramide galactosyltransferase and cerebroside sulfotransferase, the two enzymes required for sulfatide synthesis. In CHO cells synthesizing galactosylceramide but not sulfatide, galactosylceramide was also strongly reduced, suggesting an effect at the level of galactolipid synthesis. Notably, AG-205 inhibited galactosylceramide synthesis to a similar extent in wild type CHO cells and cells that lack PGRMC1 and/or PGRMC2. In vitro enzyme activity assays showed that AG-205 is an inhibitor of UDP-galactose: ceramide galactosyltransferase, but not cerebroside sulfotransferase. This study shows that PGRMC1 is only one of several targets of AG-205 and should be used with caution, especially in studies using cells synthesizing galactosylceramide and sulfatide.

## 1. Introduction

The progesterone receptor membrane component 1 (PGRMC1) is a single-pass membrane protein with a heme-binding domain that form homodimers in a heme-dependent manner [1,2]. It is a member of the membrane-associated progesterone receptor (MAPR) family [3] and is present at elevated levels in different cancer types, including breast, lung, and colon cancers [4,5,6]. Various physiological roles have been proposed for PGRMC1, which has been reviewed elsewhere [7,8]. PGRMC1 interacts and stabilizes epidermal growth factor (EGF) receptor [9]. PGRMC1 level and phosphorylation status differ between estrogen receptor α negative and positive breast cancer cells [10]. The phosphorylation status of PGRMC1 significantly influences cell migration and various metabolic functions [11]. There is a positive correlation between PGRMC1 expression level and cell viability, tumor growth and metastasis [5,6,12]. Thus, PGRMC1 is a possible target for cancer therapy and inhibitors or antagonists of PGRMC1 may have potential therapeutic value [13].

The compound AG-205 was found in a screen for ligands of the *Arabidopsis thaliana* homolog of mammalian PGRMC1 [14]. Ahmed et al. [15] showed that AG-205 binds to human PGRMC1 as well. Since then, AG-205 has been used in several studies to examine or confirm functions of PGRMC1 [9,16,17,18,19]. To our knowledge, there are no studies further examining the specificity of this compound.

**Figure 1:**
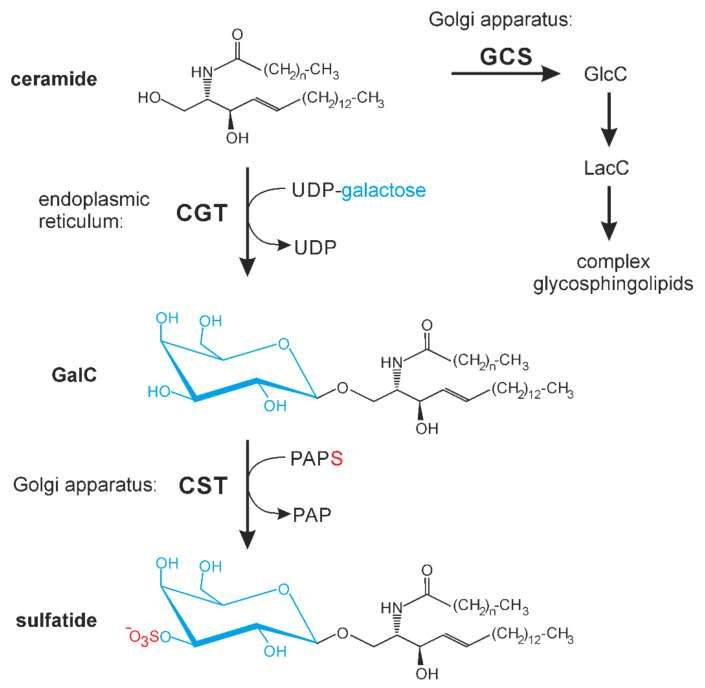
Schematic presentation of galactosylceramide (GalC) and sulfatide synthesis. While synthesis of GalC by UDP-galactose: ceramide galactosyltransferase (CGT) occurs in the endoplasmic reticulum, all other glycosphingolipids are synthesized in the Golgi apparatus. CST, cerebroside sulfotransferase; GCS, glucosylceramide synthase (EC 2.4.1.80); GlcC, glucosylceramide; LacC, lactosylceramide; PAP, 3′-phosphoadenosine 5′-phosphate; PAPS, 3′-phosphoadenosine 5′-phosphosulfate.

Several studies indicate a role of PGRMC1 in lipid metabolism. At least some cytochrome P450 enzymes (CYP450) are regulated by PGRMC1, including CYP450 enzymes involved in cholesterol biosynthesis [20]. The functional role of PGRMC1 may depend on its role in iron homeostasis [16] and/or its heme chaperone activity [20]. However, McGuire [21] recently identified a heme-independent role of PGRMC1 in stabilization of CYP450 enzymes. PGRMC1 was also found to bind Insig-1 [22], an important player in the regulation of cholesterol biosynthesis by sterol regulatory element-binding protein (SREBP) [23]. Asperger et al. [24] showed that PGRMC1 stimulates lipid raft formation and EGF receptor signaling. Besides cholesterol, lipid rafts are enriched in sphingolipids and changes in sphingolipids can also affect lipid raft function. In this context, we have identified fatty acid 2-hydroxylase (FA2H) as an interaction partner of PGRMC1 [25]. This interaction may be important for heme incorporation into the cytochrome b5 domain of FA2H, as suggested by the proposed heme chaperone activity of PGRMC1 [16,20]. In the endoplasmic reticulum, 2-hydroxylated ceramides, synthesized by FA2H, as well as non-hydroxylated ceramides, are substrates for UDP-galactose: ceramide galactosyltransferase (CGT, EC 2.4.1.47) synthesizing galactosylceramide (GalC) in the endoplasmic reticulum (Figure 1). In contrast, glucosylceramide (GlcC) and complex glycosphingolipids are synthesized in the Golgi apparatus [26]. GalC can be further modified in the Golgi apparatus by the cerebroside sulfotransferase (CST, EC 2.8.2.11), which transfers a sulfate group to the 3′-hydroxyl group of GalC to form sulfated GalC or sulfatide [27]. This sphingolipid is an abundant component of myelin in the central and peripheral nervous system [27], but is also present at high concentrations in other cells and tissues, e.g., pancreatic β-cells, immune cells, kidney [28], and certain renal cancer carcinomas [29]. Sulfatide play a role in various processes, e.g., protein sorting, cell adhesion, blood coagulation, and insulin secretion, as reviewed elsewhere [28].

In the present report, we further examined the effect of AG-205 on sphingolipid metabolism in the sulfatide synthesizing renal cancer cell line SMKT-R3 [29] and engineered Chinese hamster ovary (CHO) cell lines capable of synthesizing GalC and sulfatide [30]. We observed a specific block in the synthesis of the sphingolipid sulfatide and its precursor GalC at low µM concentrations of AG-205. No obvious changes in other membrane lipids were observed. Further experiments showed that PGRMC1 and PGRMC2 were not required for this effect and we demonstrated inhibition of GalC synthesizing enzyme CGT by AG-205.

## 2. Materials and Methods

### 2.1. Reagents

AG-205 (cis-2-[[1-(4-Chlorophenyl)-1*H*-tetrazol-5-yl]thio]-1-(1,2,3,4,4a,9b-hexahydro-2,8-dimethyl-5*H*-pyrido[4,3-*b*]indol-5-yl)-ethanone) (cat# 6242) was obtained from Bio-Techne (Minneapolis, MN, USA), dissolved in DMSO (cat# 1.02931, Merck, Darmstadt, Germany) at a concentration of 10 mM, and stored in aliquots at −20 °C. Brefeldin A (BFA) (cat# B6542) was purchased from Sigma-Aldrich (St. Louis, MO, USA), dissolved in ethanol at a final concentration of 5 mg/mL, and stored at −20 °C. MTT (3-[4,5-dimethylthiazol-2-yl]-2,5-diphenyltetrazolium bromide) (cat# M5655) was purchased from Sigma-Aldrich, dissolved in water (10 mg/mL), and stored at −20 °C. Chloroform (cat# 7331) and methanol (cat# AE71) were obtained from Carl Roth (Karlsruhe, Germany). The following lipid standards were purchased from Sigma-Aldrich (St. Louis, MO, USA): cholesterol (cat# C8667), galactosylceramide (cat# C4905), phosphatidylcholine (cat# P3556), sphingomyelin (cat# S7004), and sulfatides (cat# S1006). C16 lactosylceramide (d18:1/16:0) (cat# 860576) and 1,2-dilauroyl-sn-glycero-3-phosphoethanolamine (cat# 850702) were from Avanti Polar Lipids (Alabaster, AL, USA). All chromatographies were performed using silica gel 60 high-performance thin-layer chromatography (HPTLC) plates (cat# 1.05641, Merck, Darmstadt, Germany).

### 2.2. Cell Lines

SMKT-R3 cells [31] were maintained in DMEM/GlutaMAX medium (cat# 31966, Thermo Fisher, Waltham, MA, USA) supplemented with 9% fetal calf serum (cat# 10437028, Thermo Fisher, Waltham, MA, USA) and penicillin/streptomycin (cat# 15140122, Thermo Fisher, Waltham, MA, USA). CHO-K1 [32], CHO-Lec8 cells [33], CHO-K1 cells stably expressing the rat UDP-galactose: ceramide galactosyltransferase gene *Ugt8* (CHO-CGT), CHO-CGT cells stably expressing the murine 3′-phosphoadenosine 5′-phosphosulfate (PAPS): cerebroside sulfotransferase gene *Gal3st1* (CHO-Sulf) [30], and CHO-K1 cells deficient in PGRMC1 and/or PGRMC2 [34] were grown in DMEM: Nut Mix F12 (1:1) (cat# 21331, Thermo Fisher, Waltham, MA, USA) supplemented with 2 mM glutamine (cat# A2916801, Thermo Fisher, Waltham, MA, USA), 4% fetal calf serum, and penicillin/streptomycin. CHO-K1 cells deficient in PGRMC1 and/or PGRMC2 were stably transfected with pcDNA3-CGT. For transfection, 2.5 µg plasmid DNA, 2.5 PLUS reagent, and 7 µL lipofectamine LTX reagent (cat# 15338100, Thermo Fisher, Waltham, MA, USA) were diluted in OptiMEM medium (cat# 31985070, Thermo Fisher, Waltham, MA, USA) and added to sub-confluent cells in one well of a 6-well plate. Stable clones were selected by adding 750 µg/mL G418 (cat# G8168, Sigma-Aldrich, St. Louis, MO, USA) into the medium 48 h after transfection for two weeks. Individual clones obtained were tested for presence of CGT protein by Western blot analysis and for GalC synthesis by lipid extraction followed by thin-layer chromatography (TLC), as described below (Section 2.5).

### 2.3. Treatment of Cell Lines

Cells were treated with AG-205 dissolved in DMSO (between 1 and 10 µM, as indicated) or DMSO (as control). In some experiments, cells were treated with 5 µg/mL BFA. Reagents were added directly into culture medium containing fetal calf serum.

### 2.4. MTT Cell Viability Assay

Cells were seeded in 96-well plates (20,000 cells/well) and 24 h later treated with increasing concentrations of AG-205 (or only DMSO) for 24 h. Thereafter, cells were incubated with 0.5 mg/mL of MTT reagent for 3 h, with the medium carefully removed and cells lysed in 4 mM HCl (cat# 9277, Carl Roth, Karlsruhe, Germany) and 0.1% NP40 (cat# 74385, Sigma-Aldrich, St. Louis, MO, USA) in 2-propanol (cat# 6752, Carl Roth, Karlsruhe, Germany). Absorbance was measured at 590 nm (with 620 nm as reference wavelength) using an Infinite 200 Pro plate reader (Tecan, Männedorf, Switzerland).

### 2.5. Lipid Extraction and Thin-Layer Chromatography

Lipid extracts were prepared according to the protocol published by Bligh and Dyer [35]. Alkaline methanolysis of glycerolipids was performed as described by Bodennec et al. [36]. Lipids were dried in a SpeedVac vacuum concentrator, dissolved in chloroform/methanol (1:1), and loaded onto HPTLC plates. Lipid standards were loaded onto adjacent lanes of the same HPTLC plate. Chromatograms were developed in the solvent mixture chloroform/methanol/water (70:30:4; *v*/*v*/*v*) or twice in the solvent mixture chloroform/methanol/water (144:25:2.8; *v*/*v*/*v*) using a horizontal developing chamber (CAMAG, Muttenz, Switzerland). Lipids were visualized by spraying plates with 625 mM cupric sulfate (cat# C1297, Sigma-Aldrich) in 8% (*v*/*v*) phosphoric acid (cat# 9079, Carl Roth, Karlsruhe, Germany) followed by heating to 180 °C for 5 min [37]. Immediately after stained plates had been cooled to room temperature, they were scanned using a flatbed scanner (Epson Perfection V700 Photo) and the intensity of lipid bands was quantified using the program AIDA (Elysia-Raytest, Straubenhardt, Germany).

### 2.6. Metabolic Labelling of Cells

Cells were treated with AG-205, BFA or the solvent only (DMSO or ethanol) in complete medium with serum and metabolically labelled for 6 h by adding [1-^14^C]-d-galactose (cat# ARC0117, Hartmann Analytic, Braunschweig, Germany) or [1-^14^C]-l-serine (cat# ARC0374, Hartmann Analytic, Braunschweig, Germany) (2 or 5 µCi/well in 6-well plates) followed by lipid extraction, as described above (Section 2.5). Dried lipids were dissolved in chloroform/methanol (1:1) and lipid extracts were applied to HPTLC plates. Chromatograms were developed as described above (Section 2.5) except that a vertical developing chamber was used. Radioactivity was detected using Bioimaging screens and a Fuji BAS 1800II Bioimage Analyzer (Fujifilm, Tokyo, Japan), with the intensity of lipid bands quantified using AIDA software.

### 2.7. Western Blot Analysis

SDS-PAGE and Western blotting were performed as described previously [38]. The following antibodies were used in this study: rabbit anti-CGT (dilution 1:4000; kind gift from Gerrit van Meer) [39], rabbit anti-α-tubulin (dilution 1:20,000; cat# 600-401-880, Rockland Immunochemicals, Limerick, PA, USA), and peroxidase-conjugated goat anti-rabbit IgG (dilution 1:20,000; cat# 111-035-003, Jackson ImmunoResearch Laboratories, West Grove, PA, USA). Bound secondary antibodies were detected by enhanced chemiluminescence using Pierce ECL Western blotting substrate (Thermo Fisher, Waltham, MA, USA) and a CCD camera system (Fusion Solo X; Vilber Lourmat, Eberhardzell, Germany).

### 2.8. Indirect Immunofluorescence

Cells on glass coverslips were washed once with phosphate-buffered salt solution (PBS: 10 mM sodium phosphate pH 7.4, 145 mM sodium chloride, 5 mM potassium chloride) and fixed for 10 min in 4% paraformaldehyde in PBS. After washing three times with PBS, non-specific binding sites were blocked with 1% bovine serum albumin, 0.5% NP-40 in PBS for one hour. Incubation with primary antibodies was performed overnight at 4 °C in blocking solution. Primary antibodies used were rabbit anti-GM130 (dilution 1:200; cat# ab52649, Abcam, Cambridge, UK) and mouse anti-calreticulin (dilution 1:200; cat# 612136, BD Biosciences, Heidelberg, Germany). Secondary antibodies were Cy3-conjugated goat anti-rabbit IgG (dilution 1:400; cat# 111-165-144, Jackson ImmunoResearch Laboratories, West Grove, PA, USA) and Cy2-conjugated goat anti-mouse IgG (dilution 1:400; cat# 115-225-146, Jackson ImmunoResearch Laboratories, West Grove, PA, USA). Specimens were mounted in ProLong Diamond Antifade Mountant (Thermo Fisher, Waltham, MA, USA) and viewed using an Axiovert M200 microscope (Carl Zeiss, Jena, Germany).

### 2.9. Cerebroside Sulfotransferase (CST) and SULT1A1 Expression and Purification

Human CST (gene name: *GAT3ST1*) lacking the N-terminal transmembrane domain was expressed as fusion protein with protein A, as previously described for the murine enzyme [30]. The human *GAL3ST1* cDNA was cloned by PCR with Phusion DNA polymerase (Thermo Fisher, Waltham, MA, USA) using the oligonucleotides ATTAGAATTCTGCCGGCCTGGCCTCCA, GTTAGGTCTCGAATTGGCTCACCACCGCAGGAAATCGC and cDNA synthesized from total RNA isolated from SMKT-R3 cDNA using standard procedures. The protein A-tagged CST enzyme was purified by affinity chromatography via IgG-sepharose (cat# 17-0969-01, GE Healthcare, Chicago, IL, USA) using standard procedures [40]. Rat Sult1a1 (EC 2.8.2.1) cDNA was synthesized using oligonucleotides CCTTGGTTCCCAGTATAGCC, ATGGAGTTCTCCCGTCCAC and a rat brain cDNA. A pQE80L (Qiagen, Hilden, Germany) expression plasmid for hexahistidine-tagged mutant SULT1A1(K65E/R68G) was generated by site-directed mutagenesis using oligonucleotides CACTCCTCTAGCTTGCCACCCTGATAGATCATATCCAGGA and TGGCGGCGCCCCCATCTATGCCCGG-GTACCCTTCCT, as described [41], while mutant SULT1A1 was expressed in *E. coli* BL21(DE3) (Thermo Fisher) and affinity purified using HisPur cobalt resin (cat# 89964, Thermo Fisher, Waltham, MA, USA), as described [42]. Protein concentrations were determined using the DC protein assay (Bio-Rad Laboratories, Hercules, CA, USA).

### 2.10. Cerebroside Sulfotransferase Assay

A coupled fluorescence sulfotransferase assay using SULT1A1(K65E/R68G) sulfotransferase to regenerate 3′-phosphoadenosine 5′-phosphosulfate (PAPS; cat# A1651, Sigma-Aldrich) from 3′-phosphoadenosine 5′-phosphate with 4-methylumbelliferyl sulfate (MUS; cat# M7133, Sigma-Aldrich, St. Louis, MO, USA) as substrate was used for CST enzyme activity measurements, as described in detail elsewhere [43]. Briefly, 20 µg GalC and 0.1 µL Triton X-100 dissolved in chloroform/methanol (2:1; *v*/*v*) were air-dried in a 96-well plate. After the addition of 50 µL reaction mixture (5 mM 2-mercaptoethanol, 4 mM MUS, 10 µM PAPS, and AG-205 in 100 mM potassium phosphate pH 7.0) and 1 µg SULT1A1 enzyme, plates were pre-incubated for 20 min at 37 °C. Reaction was started by the addition of 1 µg CST enzyme and monitored using an Infinite 200 Pro plate reader (Tecan). Excitation wavelength was 360 nm, with an emission wavelength of 460 nm. AG-205 was added to a final concentration of 10 or 50 µM.

### 2.11. Ceramide Galactosyltransferase (CGT) Assay

CGT enzyme (gene name: *Ugt8*) activity was determined as described previously [44] using a microsomal membrane fraction isolated from CHO-CGT cells as enzyme source. Reaction mixtures (50 µL) contain 30 µM C_6_-ceramide (Biomol, cat# Cay62525; Cayman Chemicals, Ann Arbor, MI, USA) and 10 µM [1-^14^C]-uridine 5’-phosphate galactose (UDP-galactose) (cat# ARC3472, Hartmann Analytic, Braunschweig, Germany). Reactions were started by the addition of 140 µg microsomal membranes and incubated for 30 min at 37 °C. After stopping the reaction by the addition of 1 mL chloroform/methanol (2:1; *v*/*v*) and 0.2 mL 150 mM NaCl, the organic phase was dried, with lipids dissolved in a small volume of chloroform/methanol (1:1; *v*/*v*) and loaded onto HPTLC plates. Chromatograms were developed in chloroform/methanol/water (70:30:4; *v*/*v*/*v*), dried, and exposed to Bioimaging screens. Screens were read using a Fuji BAS 1800II Bioimage Analyzer (Fujifilm, Tokyo, Japan) and signal intensities calculated using the program AIDA (Elysia-Raytest, Straubenhardt, Germany).

### 2.12. Statistics

Data are shown as individual data points together with the mean and standard deviation. Data were analyzed using a two-tailed Student’s *t*-test or one-way ANOVA and appropriate post hoc tests (Tukey HSD or Dunnett test), with a significance level of 0.05. Calculations were performed with Microsoft Excel 2016 or Statistica 6.0.

## 3. Results

### 3.1. AG-205 Specifically Reduces Sulfatide Level in SMKT-R3 Cells

**Figure 2:**
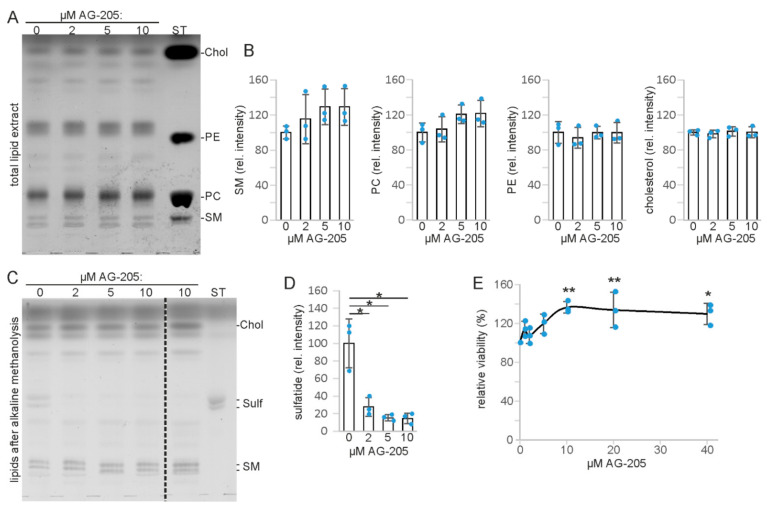
Lipid analysis and cell viability assay of SMKT-R3 cells treated with AG-205. (**A**) TLC analysis of total lipid extracts. Note that the phosphatidyl ethanolamine (PE) standard migrated significantly slower compared to PE in the cell lipid extracts because of the short chain length of the standard lipid. (**B**) Densitometric quantification of sphingomyelin (SM), phosphatidylcholine (PC), phosphatidylethanolamine (PE), and cholesterol (Chol). (**C**) TLC analysis of lipids obtained after alkaline methanolysis of glycerolipids to identify sphingolipids that co-migrate with glycerophospholipids. All lanes shown are from the same TLC plate, though several irrelevant lanes were removed (indicated by the dotted line). Lipid sample in lane 5 were from cells treated with another AG-205 batch. Position of cholesterol (Chol) and sphingomyelin (SM) in this TLC were deduced from the known Rf values. (**D**) Densitometric quantification of sulfatide (Sulf) (*n* = 3). * *p* < 0.05 (one-way ANOVA with post hoc Tukey HSD test). (**E**) MTT cell viability assay. * *p* < 0.05, ** *p* < 0.01 (one-way ANOVA with post hoc Dunnett test). Data show the relative cell viability with DMSO-treated cells (0 µM AG-205) set to 100% (*n* = 3). ST, lipid standards. Data are shown with the mean ± SD (*n* = 3 independent experiments).

In our experiments, we used AG-205 at concentrations at or below the concentrations known to inhibit PGRMC1 activity [15]. When SMKT-R3 cells, a human renal cancer cell line synthesizing the sphingolipid sulfatide [45], were treated with increasing concentrations of AG-205 (2 to 10 µM), no significant changes in major lipid classes were observed when total lipid extracts were examined by thin-layer chromatography (TLC) (Figure 2A). Densitometric quantification revealed no significant changes of sphingomyelin, phosphatidylcholine, phosphatidylethanolamine, and cholesterol content (Figure 2B). However, when sphingolipids were analyzed by TLC after alkaline methanolysis of glycerolipids (Figure 2C), we observed a specific reduction of sulfatide in cells treated with AG-205 (Figure 2D). GalC, the precursor of sulfatide (Figure 1), was hardly detectable, suggesting that the GalC level may limit sulfatide synthesis in these cells, in line with a previous report [29]. To test for a possible toxic effect of AG-205, SMKT-R3 cells were treated with up to 40 µM AG-205 for 24 h followed by MTT assay to evaluate cell viability (Figure 2E). AG-205 appeared not to be toxic, but rather increased cell viability significantly (one-way ANOVA with post hoc Dunnett test) at a concentration of 10 µM or higher. Whether this unexpected finding is a result of the sulfatide reducing activity of AG-205 was not examined further in the present study.

### 3.2. AG-205 Specifically Reduces Sulfatide and GalC Synthesis in CHO Cell Lines

**Figure 3:**
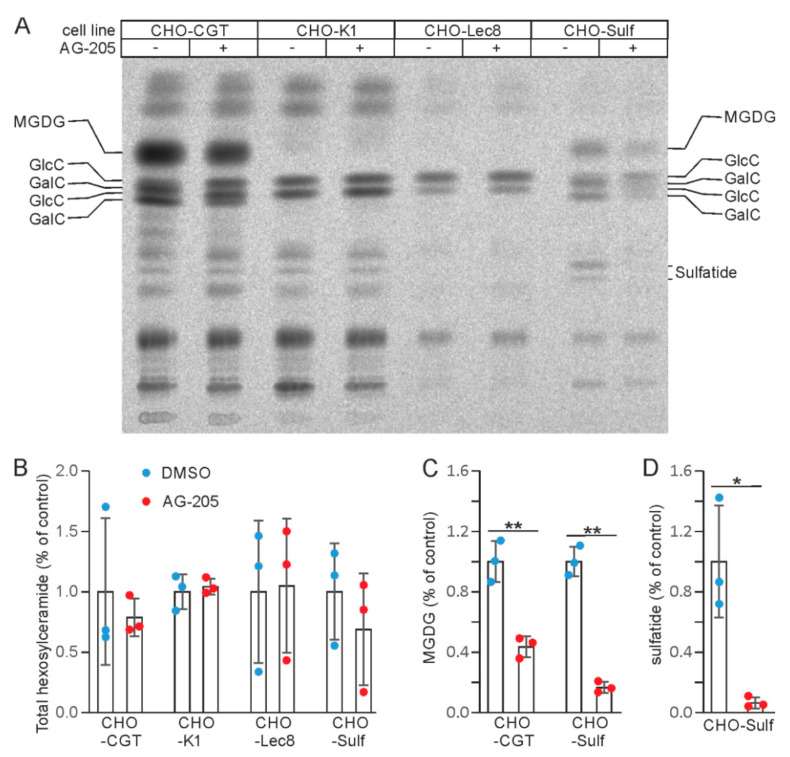
TLC analysis of CHO cells treated with AG-205. (**A**) Different CHO cell lines were treated with 10 µM AG-205 (+) or the solvent DMSO (-). Cells were metabolically labelled with [^14^C]-galactose (CHO-CGT and CHO-K1: 5 µCi; CHO-Lec8 and CHO-Sulf: 2 µCi) for 6 h and lipid extracts analyzed by TLC. CHO-CGT, CHO-K1, CHO-Lec8, CHO-Sulf. Note the shift of hexosylceramide in CHO-CGT cells and CHO-Sulf cells, which is caused by the partial replacement of GalC by GlcC, and the strong reduction of sulfatide and monogalactosyl diacylglycerol (MGDG) in CHO-Sulf cells. Note GalC, GlcC, and sulfatide migrate as duplicate bands, which represents GalC/GlcC/sulfatide with different acyl chain lengths. GalC and GlcC were identified based on the fact that CHO-K1 and CHO-Lec cells synthesize only GlcC, whereas the major hexosylceramide in CHO-CGT and CHO-Sulf cells is GalC. Position of MGDG and sulfatide were identified based on the known Rf values of these lipids and their absence in Lec8 CHO cells, which are unable to synthesize galactolipids because of a UDP-galactose transporter deficiency. (**B**) The total hexosylceramide (GalC + GlcC) levels were not significantly different between DMSO-treated controls and AG-205 treated cells. Because GlcC and GalC were not well separated, it was not possible to quantify these lipids separately. (**C**) MGDG levels were significantly reduced in AG-205-treated CHO-CGT and CHO-Sulf cells. (**D**) Sulfatide levels were significantly reduced in AG-205-treated CHO-Sulf cells. Data shown in (**B**–**D**) were normalized to the mean of the DMSO treated controls. Data are shown with the mean ± SD (*n* = 3). Asterisks indicate significant differences: * *p* < 0.05, ** *p* < 0.01 (*t*-test).

In order to examine whether AG-205-induced reduction of sulfatide content was a cell type-specific effect and to further explore the molecular mechanism leading to reduced sulfatide synthesis, we analyzed lipid synthesis in CHO-K1 cells and CHO cell lines stably transfected with UDP-galactose: ceramide galactosyltransferase (*Ugt8* encoding CGT) (CHO-CGT cells) and PAPS: cerebroside sulfotransferase (*Gal3st1* encoding CST) (CHO-Sulf cells) expression plasmids. Cells were treated with 10 µM AG-205 or the solvent DMSO for 6 h and metabolically labelled with [^14^C]-galactose. Lipids were analyzed by TLC (Figure 3). Similar results were obtained when CHO-CGT cells were metabolically labelled with [^14^C]-serine (Supplementary Figure S1).

No changes in lipid composition were observed in the parental CHO-K1 cells, which do not synthesize GalC, or the glycosylation mutant line CHO-Lec8, which is deficient in UDP-galactose transporter [33]. In contrast, CHO-Sulf cells showed a strong reduction in sulfatide synthesis, similar to SMKT-R3 cells. In addition, GalC was strongly reduced and GlcC increased in CHO-Sulf cells, as well as in CHO-CGT cells. Monogalactosyldiacylglycerol (MGDG), another product of CGT, was also reduced in CHO-Sulf and CHO-CGT cells. These results suggest that reduced sulfatide synthesis in CHO-Sulf and SMKT-R3 cells may be secondary to inhibition of GalC synthesis. Reduced GalC synthesis in the endoplasmic reticulum likely leads to an increased transfer of ceramide to the Golgi apparatus and GlcC synthesis. Taken together, the above results indicate a cell-type independent and specific reduction of CGT-dependent galactolipid synthesis by AG-205.

### 3.3. AG-205 Induced Inhibition of GalC Synthesis Is Independent of Pgrmc1 and Pgrmc2 Expression

**Figure 4:**
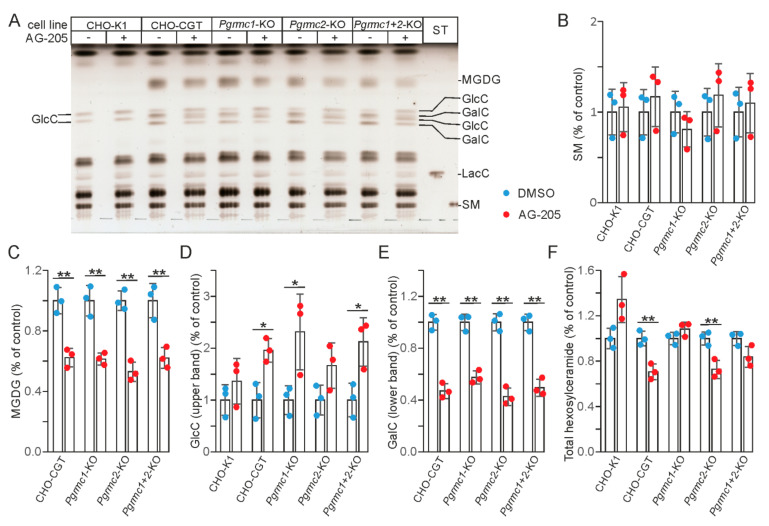
AG-205 treatment of CHO-CGT cells deficient in PGRMC1 and PGRMC2. (**A**) CHO-K1 and CHO-CGT cells lacking functional *Pgrmc1* and/or *Pgrmc2* genes were treated with 10 µM AG-205 (+) or the solvent DMSO (−) for 48 h. Total lipids were isolated and separated by HPTLC. GalC, galactosylceramide; GlcC, glucosylceramide; LacC, lactosylceramide; MGDG, monogalactosyldiacylglycerol; SM, sphingomyelin. GalC and GlcC were identified based on the fact that CHO-K1 synthesize only GlcC, whereas the major hexosylceramide in CHO-CGT cells is GalC. MGDG was identified based on the known Rf value of this lipid and its absence in CHO-K1 cells. Lipids were quantified by densitometry. (**B**) Quantification of sphingomyelin. (**C**) Quantification of MGDG. MGDG was undetectable in CHO-K1 cells. Because GlcC and GalC bands were not fully separated, total GlcC and total GalC levels could not be determined. Therefore, only the upper GlcC and lower GalC bands were quantified. (**D**) Quantification of the upper GlcC band. (**E**) Quantification of the lower GalC band. (**F**) Quantification of total hexosylceramide (GlcC + GalC). Data are shown together with the mean ± SD (*n* = 3). Asterisks indicate significant differences: * *p* < 0.05, ** *p* < 0.01 (*t*-test).

To test the hypothesis that AG-205 dependent inhibition of GalC synthesis is mediated by PGRMC1, or alternatively by the related protein PGRMC2, we stably transfected CHO cells lacking PGRMC1 and PGRMC2 separately or in combination [34] with a CGT expression plasmid and selected clones with a comparable GalC level for the experiments. Unexpectedly, we observed that AG-205 reduced GalC (and MGDG) synthesis and increased GlcC levels to a similar extent in all CHO-CGT clones independent of the presence of functional *Pgrmc1* and/or *Pgrmc2* genes (Figure 4). Thus, reduced GalC synthesis in the presence of AG-205 is not the result of PGRMC1 or PGRMC2 inhibition.

**Figure 5:**
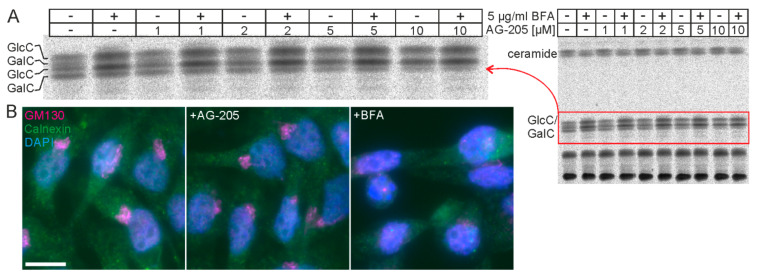
BFA and AG-205 treatment. (**A**) CHO-CGT cells were treated with 5 µg/mL BFA (+) and increasing concentrations of AG-205 (0 to 10 µM) for 16 h and then metabolically labelled with [^14^C]-serine for 6 h. Isolated lipids were separated by TLC. Only the region containing hexosylceramides is shown. (**B**) CHO-CGT cells were treated with AG-205 (10 µM), BFA (5 µg/mL), or solvents (DMSO and ethanol) for 18 h followed by immunofluorescence staining of the Golgi marker GM130 (magenta) and endoplasmic reticulum marker calreticulin (green). Nuclei were stained with DAPI (blue). Note the characteristic Golgi staining pattern of GM130 in control cells (left panel) and AG-205 treated cells and its absence in BFA-treated cells. Scale bar, 20 µm.

Because we have previously shown that AG-205 induces large vesicles, likely derived from endosomes, again independently from *PGRMC1* and *PGRMC2* expression [34], we examined whether AG-205′s effect on vesicle transport may disassemble the Golgi apparatus leading to a redistribution of Golgi resident proteins into the endoplasmic reticulum. This could potentially also explain reduced GalC and increased GlcC synthesis. In fact, treating CHO-CGT cells with brefeldin A (BFA), which blocks vesicular transport from the endoplasmic reticulum to the Golgi apparatus, increased GlcC, as expected (Figure 5A). However, AG-205 led to a more pronounced reduction in GalC (Figure 5A) and, more importantly, the structure of the Golgi apparatus appeared not to be affected by AG-205 (Figure 5B). We therefore concluded that reduced GalC synthesis in the presence of AG-205 was most likely not the result of a disassembly of the Golgi apparatus and redistribution of Golgi resident enzymes to the endoplasmic reticulum, although we did not specifically test the subcellular distribution of GlcC synthase.

### 3.4. AG-205 Inhibits UDP-Galactose: Ceramide Galactosyltransferase (CGT)

**Figure 6:**
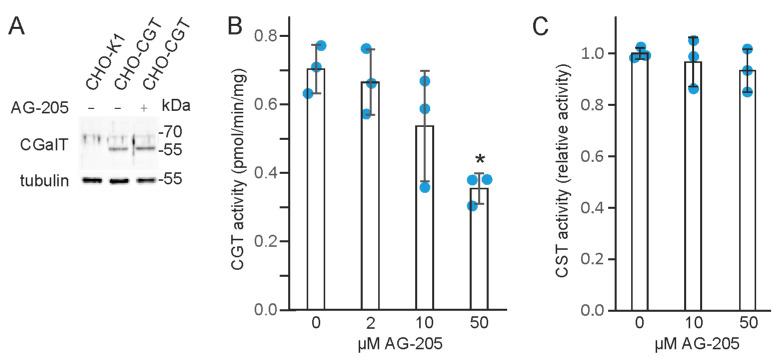
AG-205 inhibits CGT enzyme activity. (**A**) Western blot analysis of CGT in CHO-CGT cells treated with 10 µM AG-205 (or DMSO as control). Untreated CHO-K1 cells served as negative control. The blot was reprobed with α-tubulin antibody to control for equal loading. (**B**) CGT enzyme activity assay (*n* = 3). Activity was significantly inhibited by 50 µM AG-205 (one-way ANOVA [F(3,8) = 7.089 (*p* = 0.012) with post hoc Dunnett test (* *p* = 0.008)]. (**C**) CST enzyme activity assay (*n* = 3). Relative CST activity is shown (mean of control set to 100%). No significant reduction of CST activity in the presence of AG-205 was observed using one-way ANOVA [F(2,6) = 0.604 (*p* = 0.577)].

Western blot analysis showed that AG-205 treatment did not reduce CGT protein concentration in CHO-CGT cells (Figure 6A). In vitro CGT enzyme activity assays, however, demonstrated a significant inhibition of CGT activity by 50 µM AG-205 (Figure 6B). In contrast, CST enzyme activity was not significantly affected by AG-205 (Figure 6C). We therefore conclude that reduced GalC synthesis in the presence of AG-205 results from inhibition of CGT.

## 4. Discussion

We showed that the PGRMC1 antagonist AG-205 inhibits synthesis of galactolipids (GalC and MGDG) and the GalC derivative sulfatide independent of intact *Pgrmc1* (and *Pgrmc2*) gene. Results from CGT activity measurements suggest that this was caused by CGT inhibition, though we cannot rule out the possibility that other activities are additionally inhibited by AG-205. We did not examine a possible inhibition of the UDP-galactose transporter. In addition, reduced galactolipid and increased GlcC synthesis could potentially also result from a more efficient transfer of free ceramide from the endoplasmic reticulum to the Golgi apparatus. While it is known that ceramide transfer for sphingomyelin synthesis is mediated by ceramide transfer protein (CERT) [46], the pathway of ceramide transport for GlcC synthesis is not fully characterized.

The present report follows previous studies that have suggested PGRMC1-independent effects and targets of AG-205, though without identifying them. In a previous study we found that AG-205 has a significant effect on vesicular transport because it induces the formation of large to giant vacuoles (most likely derived from endosomes) in various cell lines, independent of *Pgrmc1* or *Pgrmc2* expression [34]. Furthermore, Teakel et al. [47] identified proteins significantly affected by AG-205 in the absence of PGRMC1 overexpression in a screen for PGRMC1 interacting partners. Taken together, these and the present study indicate that AG-205 is not a PGRMC1 specific inhibitor or antagonist. This may not per se exclude its usage in studies examining the role of PGRMC1, but it will be important to further characterize these other targets and the PGRMC1-independent effects caused by AG-205. In particular, cells synthesizing GalC and/or sulfatide may respond to low µM concentrations of AG-205 in a PGRMC1-independent manner. Because of their presence in lipid rafts, GalC and sulfatide can significantly affect receptor localization and signaling [48,49,50]. In particular, sulfatide is known to influence or to be critically involved in a number of cellular processes [28,51]. Treatment of cells or animals with AG-205 may significantly affect these processes, including the differentiation of oligodendrocytes and myelination, neural plasticity, insulin secretion, and immune response [28,51].

AG-205 is known to inhibit cell viability in the absence of serum [4], but to our knowledge increased cell viability in the presence of higher AG-205 concentrations have not been reported. Unexpectedly, we observed higher cell viability of SMKT-R3 cells in the presence of 10–40 µM AG-205. Although we did not examine this further, this could potentially result from the reduced sulfatide content. A proliferation stimulating and anti-apoptotic action of sulfatide deficiency in oligodendrocytes has been observed [52,53] and high sulfatide content in oligodendrocyte precursor cells is associated with reduced platelet-derived growth factor receptor association with membrane subdomains and signaling [49].

The present findings may also be of interest in the area of sphingolipidoses [54], a group of lysosomal storage diseases [55]. Specific inhibitors of CGT are of interest as potential therapeutics for Krabbe disease and metachromatic leukodystrophy [56]. AG-205 may serve as a starting compound to develop new specific CGT inhibitors.

## 5. Conclusions

Our study demonstrates that the compound AG-205 is not a specific PGRMC1 inhibitor or antagonist. Particularly in cells synthesizing GalC and sulfatide, low µM concentrations of AG-205 may provoke significant PGRMC1-independent effects.

## Supplementary Materials

The following are available online at https://www.mdpi.com/article/10.3390/cells10123520/s1, Figure S1: CGT cells metabolically labelled with [^14^C]-serine.
