# Supplementary material for: The PGRMC1 Antagonist AG-205 Inhibits Synthesis of Galactosylceramide and Sulfatide
Source: Cells. 2021 Dec 13;10(12):3520. doi: 10.3390/cells10123520 (PMC8700550; doi:10.3390/cells10123520)
Supplement: Supplementary file 1 [file cells-10-03520-s001.zip › cells-1484210-supplementary.pdf]

Supplementary Figure S1

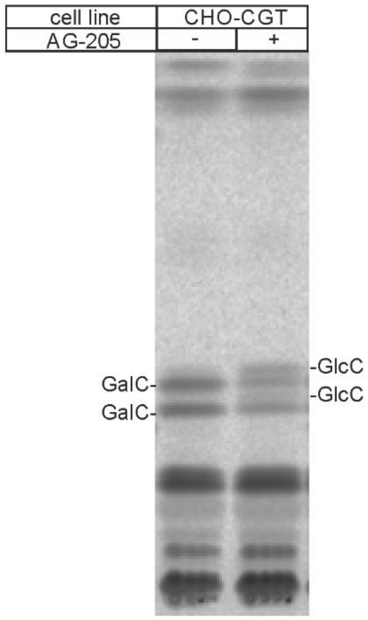

Figure S1. CGT cells metabolically labelled with [14C]-serine.

HPTLC of [14C]-serine labelled CHO-CGT cells treated with DMSO (-) or 10 µM AG-205 (+). The TLC plate was developed twice in the solvent system chloroform/methanol/water (144:25:2.8; v/v/v).
